# *Luthrodes pandava* Larvae Can Distinguish *Cycas* Leaf Quality in Cafeteria Experiments

**DOI:** 10.3390/insects16090973

**Published:** 2025-09-17

**Authors:** Thomas E. Marler

**Affiliations:** 1 Philippine Native Plants Conservation Society, Inc., Ninoy Aquino Parks and Wildlife Center, Quezon City 1101, Philippines; thomas.marler@gmail.com; 2 Cycad Specialist Group, International Union for Conservation of Nature Species Survival Commission, 1196 Gland, Switzerland

**Keywords:** cycads, Lepidoptera, Lycaenidae

## Abstract

Cycads are being endangered by several anthropogenic threats, and invasions of non-native herbivores are among those threats. Various country invasions by the cycad blue butterfly *Luthrodes pandava* have threatened the local horticulture cycad trade and native cycad populations. Little is known about behaviors of the larval stage of this specialist herbivore, the life phase that causes damage to cycad leaf tissue. The objective here was to determine larval ability to discriminate among *Cycas* species that are known to exhibit contrasting susceptibility to *L. pandava* herbivory. Dual-choice cafeteria protocols were used whereby a single larvae was provisioned with one leaflet of minimally damaged species (low quality) and one leaflet of heavily damaged species (high quality). Consumption after an 8 h feeding cycle was measured as loss in leaflet area. Wild *L. pandava* populations derived from in situ Philippine and Thailand *Cycas* habitats preferentially fed on high quality leaflets. In contrast, invasive *L. pandava* populations from urban locations in Guam, Philippines, and Thailand exhibited no preferences in leaflet feeding choice. The findings indicated that larvae of this Lycaenidae specialist herbivore exhibit complex selection behaviors that depend on origin of the butterfly and feeding history. Discrimination capabilities of the in situ populations that evolved with a single native *Cycas* species demonstrated beneficial behaviors that appear to be lost over time by invasive populations that feed on numerous novel *Cycas* hosts within the invasive range.

## Simple Summary

The butterfly *Luthrodes pandava* is a widespread invasive species that produces larvae that feed on immature leaves of *Cycas* species. Since *Cycas* taxa are members of the group of plants known as cycads, and this plant group is the most threatened plant group that has been assessed, a greater understanding of the interactions between the host and the herbivore is needed. I evaluated the ability of *L. pandava* larvae to preferentially feed on leaves from high quality *Cycas* species. The larvae from wild populations of the butterfly obtained from Philippine and Thailand habitats that contain a native *Cycas* species were able to successfully discriminate. These larvae preferentially fed on leaflets from *Cycas* species that are considered to be high quality food. In contrast, larvae from invasion populations of the butterfly collected in urban settings fed on any *Cycas* leaflet offered as food. The beneficial behaviors exhibited by the wild butterfly populations appear to be lost over time when an urban butterfly population has access to numerous *Cycas* species for regeneration. The findings indicated that the geographic source of butterflies and feeding history influence feeding behavior.

## 1. Introduction

The global biodiversity crisis has emerged as a critical research agenda [1,2]. One approach has been to create groups of species to more fully understand extinction risks to the groups as a whole [3]. Cycads are members of an ancient group of spermatophytes, and some species have become commodities in the horticulture industry [4]. Cycads have emerged as the most threatened plant group that has been assessed globally [5,6,7,8]. Furthermore, sequential assessments have shown that the threat status to this group of plants is worsening with time [3].

Habitat loss and over-collection of wild plants comprise the greatest threats to cycads [5], but invasive species have been identified as one of the other threats that are directly related to human activities [9,10,11]. The Cycadaceae is a mono-generic family comprising 125 species [12], and two invasive arthropod herbivores have emerged as major threats to members of this family in recent years. The armored scale *Aulacaspis yasumatsui* Takagi can feed on numerous cycad species (Cycadophyta), but its ability to kill its host appears to be restricted to *Cycas* species [9]. The *Luthrodes pandava* Horsfield (Lepidoptera: Lycaenidae; formerly *Chilades pandava*) is a voracious leaf-feeding Lycaenidae butterfly that has a widespread native range [10,13,14]. Variation in the amount of *L. pandava* damage among co-mingled *Cycas* L. species is considerable, with the species which coevolved with a leaf-feeding butterfly revealing the least levels of herbivory [15].

Evidence-based conservation protocols are of paramount importance to mitigate the threats to cycad species survival [11]. Therefore, a greater understanding of the manner in which *L. pandava* threatens cycad plants and the defensive traits that the host plants utilize to confront *L. pandava* herbivory is needed. Moreover, for the endemic regions in which a native *Cycas* host co-exists with the native *L. pandava*, the ethical obligation to conserve both species presents a conundrum for decision-makers [15,16]. Very few ethology experiments have been conducted to more fully understand this bi-trophic relationship. When a *L. pandava* larvae is reared on leaflets from the most damaged *Cycas* species, a clear benefit has been demonstrated through oviposition performance of the consequent adults [17]. These authors reported that adult female *L. pandava* lifespan was increased 32% and lifetime egg production was increased more than 50% when individuals reared on leaves from heavily damaged *Cycas* species were compared with individuals reared from minimally damaged species. Similar influences on life history traits were recorded when *L. pandava* larvae diet was restricted to leaves from low quality *Zamia* species versus high quality *Cycas* species [18,19]. For the purposes of this study, the species that are heavily damaged by *L. pandava* feeding are considered high quality, and the species that are minimally damaged are considered low quality.

The host species that serves as larvae food clearly imposes an increase or decrease in adult performance of the herbivore. Therefore, discrimination capabilities would benefit the butterfly population in locations where more than one host species is available. Evaluating choices made by gravid *L. pandava* females indicated these individuals were able to discriminate among various *Cycas* species, preferentially ovipositioning on the leaves of high-quality species [20]. Interestingly, this behavior was restricted to a wild population of *L. pandava* which was derived from the endemic *Cycas nongnoochiae* K.D. Hill habitat. Females from an urban population of *L. pandava* of unknown origin and a history of feeding on numerous *Cycas* species were unable to discriminate among *Cycas* species, revealing no preferences when presented with high quality versus low quality leaves for ovipositioning. To my knowledge, the ability of *L. pandava* larvae to discriminate between high quality versus low quality *Cycas* leaves has not been studied to date.

Traditional dual-choice cafeteria feeding trials were employed here to determine the feeding behavior of *L. pandava* larvae when provisioned with leaflet tissue from one low quality *Cycas* species and one high quality *Cycas* species. The objective was to determine if individual larvae of this *Cycas* herbivore can discriminate food options. The findings are of importance for improving management decisions in botanic garden settings in which *L. pandava* has access to numerous host species grown in close proximity.

## 2. Materials and Methods

Dual-choice cafeteria trials were conducted in Guam, Philippines, and Thailand with *L. pandava* larvae derived from three in situ *Cycas* populations which had fed exclusively on their native *Cycas* host, and three urban populations of unknown origin and which had fed on several *Cycas* species located in the urban forest or garden community. The feeding trials were designed to provision one leaflet choice from a high quality *Cycas* species and one leaflet choice from low quality *Cycas* species, based on a common garden survey of *L. pandava* damage to 85 *Cycas* species [15]. The response variable calculated was amount of leaflet consumed from each leaflet during a 8 h feeding cycle. A total of 280 larvae derived from seven populations were exploited in this study.

### 2.1. Population Origins

**Figure 1:**
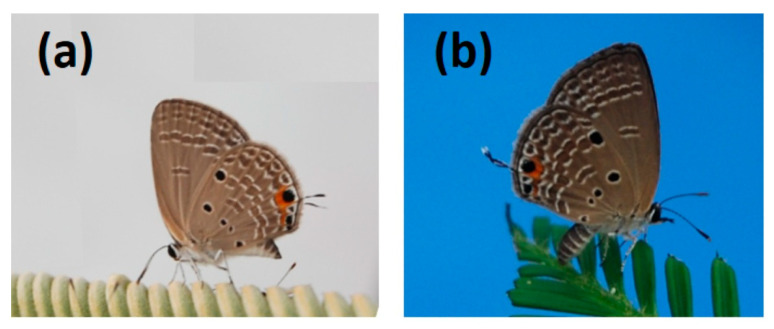
*Luthrodes pandava* adult female butterflies in Thailand. (**a**) Derived from Tak Fa from an endemic *Cycas nongnoochiae* habitat; (**b**) derived from Nong Nooch Tropical Botanical Garden.

Butterfly eggs and larvae were collected in Tak Fa, Thailand within the *C. nongnoochiae* endemic range on 18–19 June 2013. The eggs and larvae were transported to Nong Nooch Tropical Botanical Garden (NNTBG) in Chonburi, Thailand and fed *C. nongnoochiae* leaves until pupation. The Gardens’ extensive cycad collection was the source of the food. The resulting population of adults (Figure 1a) were fed honey on sponges and maintained in a communal cage ca. 1.6 m^3^ in volume. Production of larvae used in the cafeteria trials is described below.

The NNTBG’s *Cycas* germplasm supported a thriving *L. pandava* population of unknown origin (Figure 1b). This population had been feeding on numerous *Cycas* species for many years. Young *C. revoluta* leaflets containing numerous *L. pandava* eggs were collected from the Garden 13–16 July 2013 to produce the larvae as described below.

**Figure 2:**
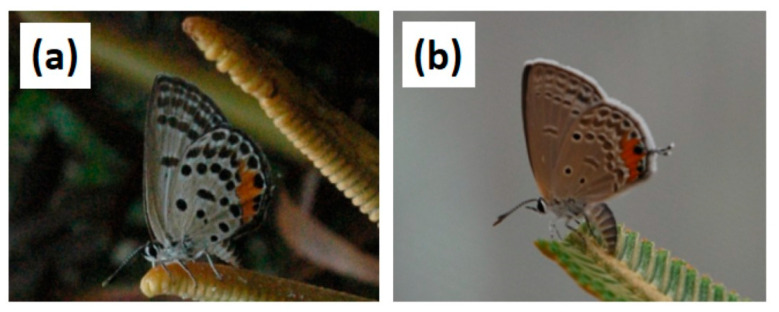
*Luthrodes pandava* adult female butterflies in the Philippines. (**a**) Derived from Mansalay, Mindoro from a *Cycas edentata* habitat; (**b**) derived from Angeles City, Pampanga from *Cycas* plants in the urban forest.

Butterfly larvae were collected in Mansalay, Mindoro, Philippines from a coastal *Cycas edentata* de Laub. population on 9 January 2019. The collections were made with permission from the owner of the copra farm that contained the plants. The trees on the farm were a component of a large *C. edentata* population that extended along the coastline in undisturbed habitats. The larvae were transported to a *Cycas* germplasm garden in Angeles City, Philippines and fed *C. edentata* leaflets until pupation. The resulting population of adults (Figure 2a) were fed honey on sponges and maintained in a communal cage ca. 1.6 m^3^ in volume. Production of larvae is described below.

The urban population of *Cycas* plants in the Angeles City urban forest supported a widespread *L. pandava* population of unknown origin (Figure 2b). This population had been feeding on the available *Cycas bougainvilleana* K.D. Hill, *C. edentata*, and *C. revoluta.* Expanding *C. revoluta* leaflets containing numerous *L. pandava* eggs were collected from *C. revoluta* plants 23–28 January 2019 to produce the larvae.

**Figure 3:**
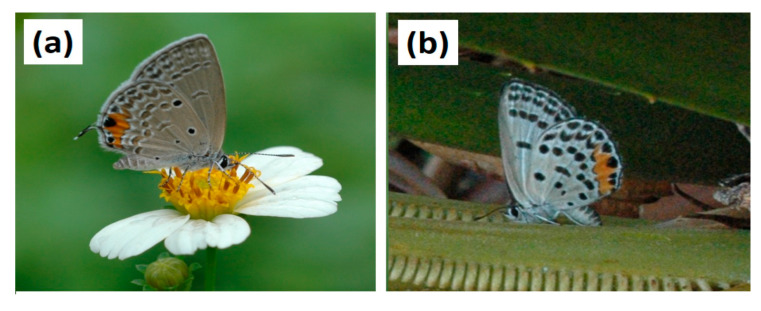
Morphology of *Luthrodes pandava* from two islands. (**a**) Invasive butterfly adult on Guam feeding on the invasive *Bidens pilosa*; (**b**) native butterfly in southern Zambales, Philippines.

The invasive *L. pandava* population on the island of Guam (Figure 3a) was used for feeding trials in 2023. Origin of this population was most likely the Philippine Archipelago [13], and it had been feeding on urban landscape *C. revoluta* and in situ *Cycas micronesica* K.D. Hill trees since the 2003 invasion [21]. Expanding leaves with numerous *L. pandava* eggs were collected directly from *C. revoluta* in the urban forest on 22–27 January 2023 and transferred to an open-air laboratory setting at the University of Guam to produce the larvae for the cafeteria trials.

Butterfly larvae were collected in Silanguin Cove, Zambales, Philippines at the southern end of the known *Cycas zambalensis* Madulid & Agoo endemic range on 7 May 2024. The collections were made with permission from plants located in private beach resort landscapes. The larvae were transported to the *Cycas* germplasm garden in Angeles City, Philippines and fed *C. zambalensis* leaves until pupation. The resulting population of adults (Figure 3b) were fed honey on sponges and maintained in a communal cage ca. 1.6 m^3^ in volume for the production of larvae.

Expanding *C. revoluta* leaves containing numerous *L. pandava* eggs were collected 21–27 May 2024 from the urban population of plants in Angeles City. A population of *L. pandava* larvae derived from these eggs was reared using the same methods as the Zambales population.

For every population of caged adults described, partially expanded *C. revoluta* leaves were offered to the adult females for ovipositioning, and the resulting larvae were initially fed ad libitum with twice daily additions of fresh *C. revoluta* leaves. The timespan from egg to adult can be as short as 11–12 d, and each female adult is capable of producing 21–27 eggs per day [17]. Therefore, producing a sizable population of growing *L. pandava* larvae was a rapid endeavor. For every urban population in which eggs were collected, the resulting larvae were reared on *C. revoluta* leaf tissue at the same time as the wild populations.

### 2.2. Rearing and Handling Larvae

The prescribed age of the larvae and the window of time that feeding was allowed for the cafeteria trials were explored by preliminary studies. Tissue loss during feeding by small larvae can be minimal, as the 1st instar is less than 1.5 mm in length. Moreover, the smallest larvae feed on leaflet surfaces and the larger larvae feed on leaflet margins, so quantifying tissue loss following feeding is difficult for the small larvae. In contrast, leaflet tissue loss during feeding by 4th instar larvae nearing pupation is extensive and tissue loss is rapid. In order to exploit a median speed of herbivory, all cafeteria assays were conducted when larvae reached 6–7 mm in length during the 2nd instar. This size is about half of maximum size of a mature 4th instar larvae. This larvae length was achieved in 5–6 days for the urban populations and 6–7 days for the wild populations. Initial feeding trials were 24 h and 18 h in duration, but 100% of the leaflet tissue was consumed for each of these durations. Feeding trials of 12 h and 10 h enabled some remaining uneaten tissue, but more than half of the available tissue was consumed. Therefore, the potential for no-choice feeding for the latter portion of the window of time disallowed feeding for this length of time. The preliminary feeding times were used to determine that 8 h was adequate for all feeding trials because the 2nd instar larvae had consumed less than half of the available leaflet tissue within this duration. This protocol enabled each larvae to feed ad libitum while being presented with an active choice between leaflets of the two available *Cycas* species for the full duration of each 8 h trial.

Selection of the two *Cycas* species to exploit for the dual-choice trials was controlled by three factors. First, the larvae of this butterfly cannot consume mature leaflet tissue, so availability of actively expanding immature leaves is mandatory. The two species selected to provide food for each of the feeding trials was therefore restricted to locally available expanding leaves in the ex situ germplasm collections that were exploited for leaflet tissue. Second, the reported assessment of species susceptibility to *L. pandava* herbivory included 85 *Cycas* species [15]. This report included six species that exhibited the greatest level of butterfly damage, and eight species that exhibited the least level of damage. One species was selected from each of these two groups for each trial, ensuring the greatest level of contrast in level of herbivory between the two selected species. Third, in order to ensure that there was no influence of *Cycas* species in larvae feeding history on the outcomes, the two *Cycas* species offered in the feeding trials were novel substrates for the individual larvae used for the trials (food was restricted to *C. revoluta* prior to the trials) but also for the historical access to *Cycas* by each butterfly population.

**Figure 4:**
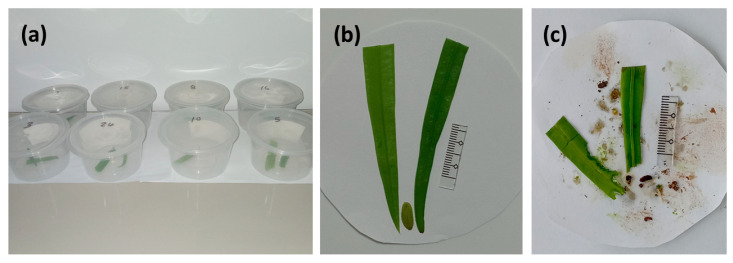
Dual-choice cafeteria feeding trials with 2nd instar *Luthrodes pandava* larvae and partially expanded leaflets from two *Cycas* species. (**a**) Vessels used for the 8 h feeding trials; (**b**) initial layout and positioning of the larvae with high-quality *Cycas micronesica* leaflet (left) and low-quality *Cycas wadei* leaflet (right); (**c**) example of appearance of the partially consumed leaflet tissue after 8 h feeding cycle. Markers on scale are 1 mm.

Larvae were selected about 1800 HR on the day prior to each trial, and all leaf material was removed from the growth chambers to force an overnight fasting period. The feeding trials were initiated 0700–0800 HR the following morning. The feeding trial containers were plastic tubs with inside basal diameter of 8.5 cm and height of 6 cm (Figure 4a). The base of each tub was covered with a moistened double layer of coffee filter paper, and the leaflets and larvae were positioned gently in the center of the paper. Each dual-choice cafeteria feeding trial was conducted with a single 2nd instar larvae positioned between two leaflet sections (Figure 4b). The leaflets were immature and rapidly expanding when selected for the offered food. Each leaflet was shortened to a maximum of 8 cm by removing the basal portion with shears. The undisturbed apex of each leaflet was positioned adjacent to each larvae, ensuring the leaflet tissue in close proximity to the larvae at the start of the feeding trials did not contain any potential induced chemicals that may have resulted from the wound on the laminae.

The layout of the trial employed four initial arrangements for each butterfly population in order to account for directional bias in the navigation [22]. Two were positioned with the leaflets oriented east–west and the more resistant leaflet was placed on the south side of the larvae for half of these and on the north side of the larvae for the other half. Two were positioned with the leaflets oriented north–south and the more resistant leaflet was placed on the east side of the larvae for half of these and the west side of the larvae for the other half. An equal number of replications were ensured for each of the four arrangements. Moreover, in order to further counter directional bias, the tubs were gently rotated 90° every 2 h during each feeding cycle.

An initial digital photograph was secured (e.g., Figure 4b), then the larvae were directly observed until feeding was initiated. This time was recorded for each replication, then each tub was loosely covered. The larvae never attempted to navigate the sides of the vessels, and remained in contact with one or both of the leaflet sections for the duration of the 8 h feeding cycles. The edge feeding behavior resulted in the two partially eaten leaflet portions remaining fully intact (Figure 4c), and each was carefully extracted with tweezers and placed onto a fresh sheet of white paper to photograph a second time. This transfer was required because the abundant frass and a change in the filter paper color due to the release of chlorophyll and frass pigments during the feeding cycle. Deleting this background noise was required to ensure unambiguous color contrast between leaflet color and background color for the image analyses (described below).

### 2.3. Dual-Choice Feeding Decisions

The feeding trials with the endemic *C. nongnoochiae* butterfly population and the resident NNTBG population were conducted 19–24 July 2013. The tubs were placed on benches under concrete slabs that allowed diffuse light but blocked the sun. A shaded maximum/minimum thermometer was installed and data were recorded daily (Table 1). The photoperiod was 12.72 h and photosynthetic photon flux density (PPFD) was recorded by placing the quantum sensor (Skye SKP200, Skye Instruments, Llandrindod Wells, Powys, UK) inside the tubs for measurements. Two species of high quality host plants were selected as *C. micronesica* and *Cycas seemanii* A. Braun. (high quality). Two species of low quality host plants were selected as *Cycas wadei* Merr. and *Cycas condaoensis* K.D. Hill & S.L. Yang (low quality). These four *Cycas* species were novel substrates for the wild population of larvae. Ten trials were conducted for each of the four *Cycas* species combinations, for a total of 40 feeding trials for each of the two butterfly populations.

The feeding trials with the Mindoro *C. edentata* butterfly population and the urban Angeles City population were conducted in an ex situ cycad germplasm garden established by the University of Guam in Barangay Sapang Bato. The tubs were placed on benches under metal roofing that allowed scattered light. The photoperiod was 11.56 h, and the species selected for the feeding trials were the high quality *C. micronesica* and the low quality *C. wadei*. These species were novel food sources for the butterflies in the trials. Forty feeding trials were conducted with the two species choices for each of the two butterfly populations.

The feeding trials with the invasive Guam butterfly population were conducted on benches under the roof overhang of an open-air laboratory under ambient conditions. The photoperiod was 11.53 h and PPFD, and the high quality species was *Cycas tansachana* K.D. Hill & S.L. Yang and the low quality species was *C. wadei*. Both of these species were new to the naturalized butterfly population on the island. A cycad research garden maintained by the University of Guam was the source of these substrates. Forty feeding trials were conducted with the two species choices.

The feeding trials with the Zambales *C. zambalensis* butterfly population and the urban Angeles City population were conducted in the same nursery setting that was used for the 2019 trials. The photoperiod was 12.99 h, and the high quality leaflets were *C. tansachana* and the low quality leaflets were *C. wadei*. Both of these species were novel food materials for the butterflies in this feeding trial. Forty feeding trials were conducted with the two species choices for each of the two butterfly populations.

### 2.4. Image and Statistical Analysis

The 600 dpi digital images were evaluated using ImageJ version 1.53 [23] to calculate individual leaflet area initially and after feeding occurred. The feeding of late 2nd instar *L. pandava* larvae is typically edge feeding if the leaflet tissue is soft enough, so there were no feeding holes to evaluate in the area calculations. The precise area of the leaflets prior to the feeding trial differed slightly, so the response variable that was considered the most accurate for comparison among all of the trials was absolute tissue area loss due to feeding. This was calculated for each leaflet by subtracting the ending area from the initial area.

The initial leaflet area data conformed to parametric analyses because of the highly prescribed manner in which the leaflets were prepared, so a standard *t*-test was employed to confirm initial leaflet area was homogeneous. The ending leaflet area data did not meet parametric prerequisites for the wild butterfly populations. As a result, the nonparametric Mann–Whitney *U* test was employed to compare the two *Cycas* species in each trial. In contrast, the ending leaflet area for the feeding trials that used the urban butterfly populations met parametric analyses requirements, so a *t*-test was employed to compare herbivory of high quality versus low quality species. For the 2013 Thailand study, each of the four two-species combinations were analyzed separately (*n* = 10). For the other three studies, there were only two species included as food offerings within each trial, so the entire 40-replication data set was analyzed. Excel Version 2506 for Microsoft 365 was employed to conduct the *t* and *U* tests.

The feeding trials were not set up to subject the wild versus urban population response variables to statistical analysis. To gain more insight on the outcomes, the means ± standard deviation for feeding efficiency (time to begin feeding) were calculated for each wild and each urban population. Similarly, the total amount of herbivory during the 8 h feeding cycle was calculated for each replication by adding the total tissue lost from both leaflets. These data were also presented as means ± standard deviation.

## 3. Results

### 3.1. Thailand

**Figure 5:**
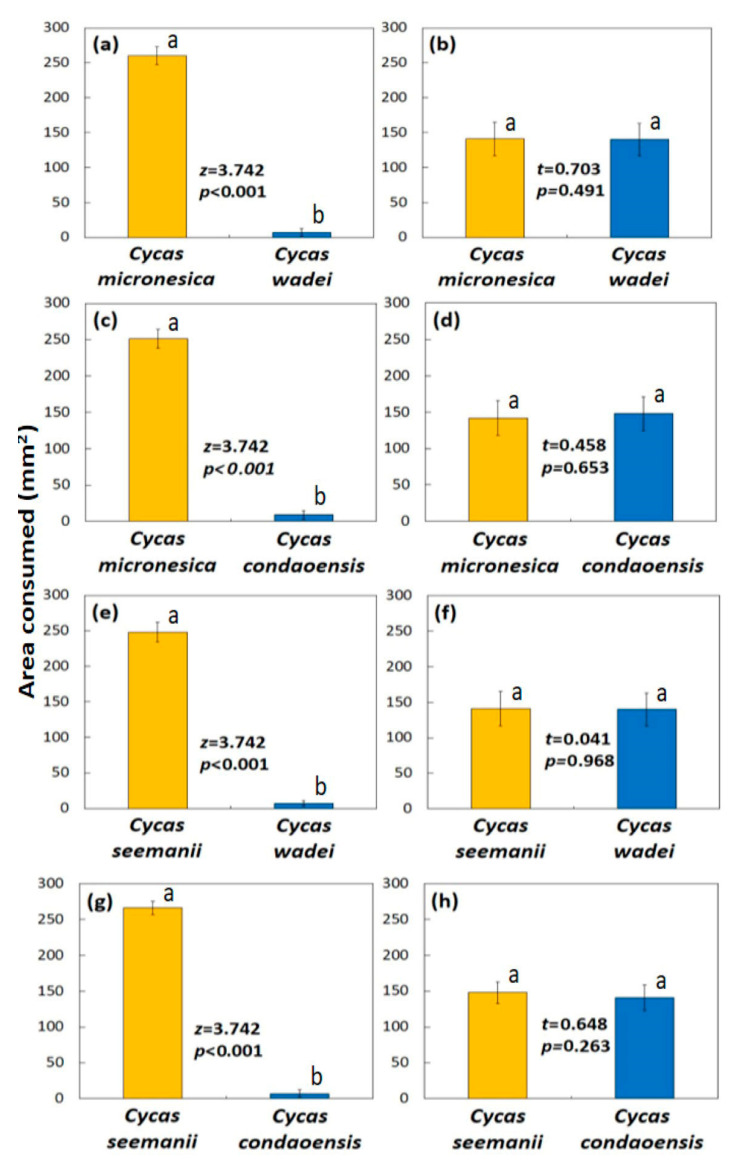
The area of leaflet tissue consumed by Thailand *Luthrodes pandava* larvae during an 8 h feeding cycle. Yellow bars depict high quality species, blue bars depict low quality species. (**a**,**c**,**e**,**g**) Wild population collected from Tak Fa; (**b**,**d**,**f**,**h**) urban population collected from Nong Nooch Tropical Botanical Garden Mean ± SD, *n* = 10. Bars within each panel with same letters do not significantly differ.

The area of *C. micronesica* and *C. wadei* leaflets that were offered to the wild Thailand butterfly larvae did not differ (*t* = 0.094, *p* = 0.926), and the larvae were given access to 477 ± 16 mm^2^ (mean ± SD; *n* = 20) for each leaflet. Feeding by these larvae began after 1.7 ± 0.9 min. These larvae consumed some low-quality *C. wadei* leaflet tissue, but the amount was only 3% of the *C. micronesica* leaflet tissue that was consumed (Figure 5a). The urban Thailand butterfly larvae were offered leaflets from the same two *Cycas* species, and the initial leaflet area did not differ (*t* = 0.452, *p* = 0.657). Initial area was 484 ± 15 mm^2^ per leaflet. Feeding began after 0.9 ± 0.2 min. Contrary to the behavior of the wild population, these urban butterfly larvae consumed similar amounts of tissue from the two *Cycas* species (Figure 5b). The urban population revealed no ability to discriminate high versus low quality substrates. Total leaflet area consumed by the wild population was 261 ± 13 mm^2^, and total area consumed by the garden population was 282 ± 16 mm^2^.

The area of *C. micronesica* and *C. condaoensis* leaflets that were offered to the wild Thailand butterfly larvae did not differ (*t* = 0.079, *p* = 0.860), and the larvae were given access to 510 ± 13 mm^2^ for each leaflet. Feeding began after 1.8 ± 0.9 min. These larvae consumed a small amount of *C. condaoensis* leaflet tissue, but the amount was only 4% of the high-quality *C. micronesica* leaflet tissue that was consumed (Figure 5c). The urban Thailand butterfly larvae were offered leaflets from the same two *Cycas* species, and the initial leaflet area did not differ (*t* = 0.331, *p* = 0.744). Initial area was 502 ± 16 mm^2^ per leaflet. Feeding began after 0.8 ± 0.2 min. These urban butterfly larvae consumed similar amounts of tissue from the two *Cycas* species (Figure 5d). The wild population consumed 258 ± 16 mm^2^ of leaflet area, and the garden population consumed 291 ± 19 mm^2^ of leaflet area.

The area of *C. seemanii* and *C. wadei* leaflets that were offered to the wild Thailand butterfly larvae did not differ (*t* = 0.178, *p* = 0.861), and the larvae were given access to 487 ± 16 mm^2^ for each leaflet. Feeding began after 2.1 ± 0.9 min. The consumption of low-quality *C. wadei* leaflet tissue by these larvae was minimal and represented about 2% of the *C. seemanii* leaflet tissue that was consumed (Figure 5e). The urban Thailand butterfly larvae were offered leaflets from the same two *Cycas* species, and the initial leaflet area was similar (*t* = 0.694, *p* = 0.497). Initial area was 489 ± 13 mm^2^ per leaflet. Feeding began after 0.7 ± 0.2 min. Leaflet tissue from the two *Cycas* species was consumed in similar amounts by these urban butterfly larvae (Figure 5f). Total leaflet area consumed by the wild population was 249 ± 13 mm^2^, and total area consumed by the garden population was 285 ± 14 mm^2^.

The area of *C. seemanii* and *C. condaoensis* leaflets that were offered to the wild Thailand butterfly larvae did not differ (*t* = 0.440, *p* = 0.665), and the larvae were given access to 517 ± 11 mm^2^ for each leaflet. Feeding began after 2.2 ± 0.8 min. The consumption of low-quality *C. condaoensis* leaflet tissue by these larvae was minimal and represented about 3% of the *C. seemanii* leaflet tissue that was consumed (Figure 5g). The urban Thailand butterfly larvae were offered leaflets from the same two *Cycas* species, and the initial leaflet area was similar (*t* = 0.169, *p* = 0.868). Initial area was 509 ± 18 mm^2^ per leaflet. Feeding began after 0.8 ± 0.2 min. The urban butterfly larvae consumed similar amounts of tissue from the two *Cycas* species (Figure 5h). The wild population consumed 268 ± 11 mm^2^ of leaflet area, and the garden population consumed 290 ± 22 mm^2^ of leaflet area.

### 3.2. Philippines

**Figure 6:**
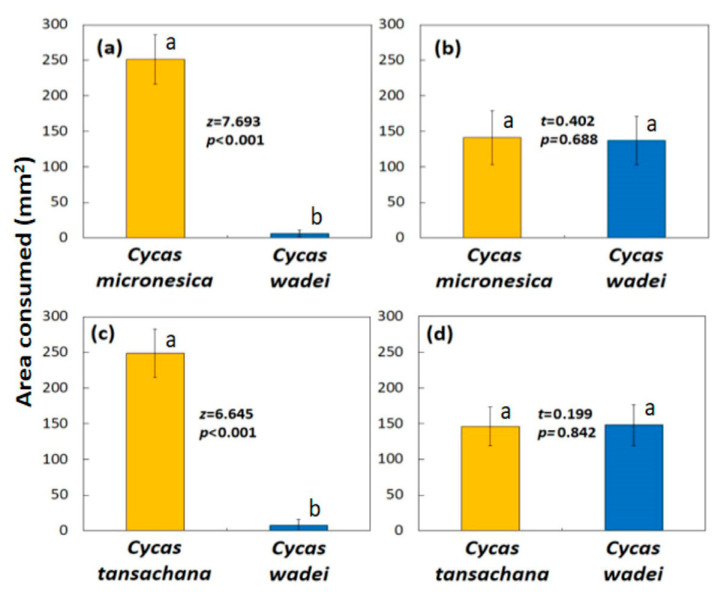
The area of leaflet tissue consumed by Philippine *Luthrodes pandava* larvae during an 8 h feeding cycle. Yellow bars depict high quality species, blue bars depict low quality species. (**a**) Wild population collected from Mindoro; (**b**) urban population collected from Angeles City; (**c**) wild population collected from Zambales; (**d**) urban population collected from Angeles City. Mean ± SD, *n* = 40. Bars within each panel with same letters do not significantly differ.

The area of *C. micronesica* and *C. wadei* leaflets that were offered to the wild Mindoro butterfly larvae did not differ (*t* = 0.598, *p* = 0.552), and the larvae were given access to 515 ± 27 mm^2^ (mean ± SD; *n* = 80) for each leaflet. Feeding began after 2.1 ± 0.9 min. These larvae avoided the low-quality *C. wadei* leaflet tissue, with herbivory in the amount of about 7% of the *C. micronesica* leaflet tissue that was consumed (Figure 6a). The urban Angeles City butterfly larvae were offered leaflets from the same two *Cycas* species, and the initial leaflet area did not differ (*t* = 0.392, *p* = 0.696). Initial area was 499 ± 18 mm^2^ per leaflet. Feeding began after 0.9 ± 0.4 min. These urban butterfly larvae consumed similar amounts of tissue from the two *Cycas* species (Figure 6b). The wild population consumed 255 ± 35 mm^2^ of leaflet area, and the urban population consumed 282 ± 29 mm^2^ of leaflet area.

The area of *C. tansachana* and *C. wadei* leaflets that were offered to the wild Zambales butterfly larvae did not differ (*t* = 0.135, *p* = 0.893), and the larvae were given access to 461 ± 21 mm^2^ for each leaflet. Feeding began after 2.1 ± 0.9 min. These larvae avoided the low-quality *C. wadei* leaflet tissue, with herbivory in the amount of about 3% of the *C. tansachana* leaflet tissue that was consumed (Figure 6c). The urban Angeles City butterfly larvae were offered leaflets from the same two *Cycas* species, and the initial leaflet area did not differ (*t* = 0.135, *p* = 0.893), and initial area was 460 ± 18 mm^2^ per leaflet. Feeding began after 0.8 ± 0.4 min. These urban butterfly larvae consumed similar amounts of tissue from the two *Cycas* species (Figure 6d). Total leaflet area consumed by the wild population was 254 ± 33 mm^2^, and total area consumed by the urban population was 295 ± 23 mm^2^.

### 3.3. Guam

**Figure 7:**
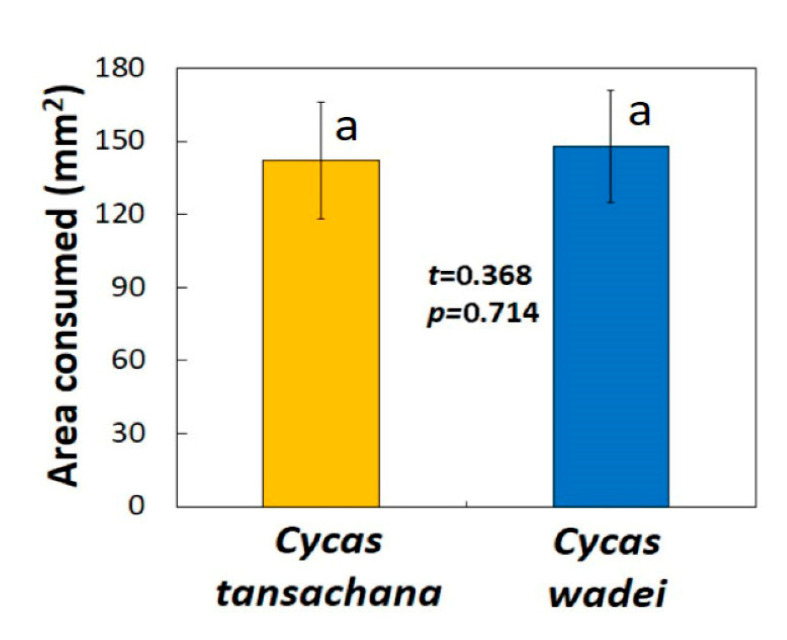
The area of leaflet tissue consumed by larvae derived from the invasive population of *Luthrodes pandava* throughout the island of Guam. Yellow bar depicts high quality species, blue bar depicts low quality species. Larvae were provided access to two *Cycas* species for 8 h. Mean ± SD, *n* = 40. Bars with same letters do not significantly differ.

Larvae derived from the invasive population of *L. pandava* throughout the island of Guam were offered leaflets from high-quality *Cycas tansachana* and from low-quality *Cycas wadei*. The initial leaflet area did not differ (*t* = 0.332, *p* = 0.741), and the larvae were presented with 453 ± 19 mm^2^ for each of the leaflets. Feeding began after 0.7 ± 0.3 min for these larvae. These butterfly larvae consumed similar amounts of tissue from the two *Cycas* species during the dual-choice feeding cycle, revealing no ability to identify differences in food quality (Figure 7). Total leaflet area consumed by these urban butterfly larvae was 309 ± 29 mm^2^.

## 4. Discussion

The current global biodiversity crisis [1,2] has fueled the need to identify groups of plants that are threatened as a means of focusing conservation research efforts. The compelling global change factors present disproportionate levels of threats to rare plants with limited numbers or restricted endemic range [24,25]. This crisis is partly fueled by invasive species, and improvements in understanding the interactions between non-native arthropod herbivores and the new hosts that are found within their invasive range are needed as part of the efforts to combat the crisis. The relevance of these issues for cycad conservation includes the voracious leaf herbivore *L. pandava* which is capable of consuming immature leaf tissues from numerous *Cycas* and *Zamia* species [15,18,19]. The butterfly therefore represents an acute threat particularly for *Cycas* species that did not coevolve with a native butterfly herbivore [15]. This threat may be manifested when the butterfly invades new geographic regions with native *Cycas* species, or when the butterfly resides in an extensive cycad germplasm garden where numerous host species are growing together. The same phenomena may occur for the *Zamia*–*Eumaeus* bi-trophic herbivory system [26].

The results herein illuminated beneficial larval discrimination capabilities of three wild *L. pandava* populations that co-evolved with a single *Cycas* host species, showcasing behaviors that enabled preferential feeding on high-quality substrates when the choices were novel, first-time choices. However, this behavior was not evident in larvae from invasive *L. pandava* populations that had been afforded a multi-species history of access to numerous *Cycas* and *Zamia* hosts within a managed botanic garden in Thailand or an invaded urban forest setting in Guam and the Philippines.

### 4.1. Host and Herbivore

Inter-generic and inter-specific differences in herbivory are clearly evident in the group of plants known as cycads, but remain equivocal and not very well understood. Some factors that may define *L. pandava* herbivory diversity have been previously discussed [27]. Active selection of one host among several available hosts may be founded in differences in nutrient content and/or palatability. Active avoidance may be founded in constitutive or induced chemical defense. Active avoidance may also be a result of structural defense. Apparency differences [28] among host species may be causal of herbivory disparity as *Cycas* leaf expansion rates vary, and these rates may translate into timing of leaf maturity. The factors which govern herbivore–host relationships in cycad biology remain obscure, and the roles of endosymbionts and sequestered biochemicals as drivers of anti-herbivory behaviors in cycads are of particular interest and deserve more study [14,29,30,31,32,33,34,35,36,37].

In the *Cycas*–*Luthrodes* herbivory system, the decision by the herbivore to avoid low quality substrates and/or seek out high quality substrates is evident in adult female oviposition choices [17]. However, the immature stages of insects possess limited mobility and their sensory systems are constrained compared to the adult stage [38]. Feeding preferences and food discrimination ability of immature individuals often do not align with those of oviposition preferences of the female parents [39,40]. The new knowledge communicated herein reveals that *L. pandava* larvae can be endowed with the same selection abilities as the female adults, corroborating robust reviews indicating butterfly larvae do possess beneficial behaviors in selecting high quality versus low quality substrates [41].

The findings presented herein also revealed that the butterfly population being studied exerted profound influences on the larvae’s choices. This study included larvae from two subspecies of *L. pandava*. The populations from Thailand conform to the subspecies *pandava* from Southeast Asia, and the wild-collected populations from the Philippines are considered the separate subspecies *vapanda* [42]. Genetic signatures have been used to indicate the most likely source of the butterfly invasion of Guam is the Philippine archipelago [13]. Based on wing coloration, the urban population of *L. pandava* in the Philippines (Figure 2b) is more closely aligned with the Thailand population (Figure 1) than the wild-type in the Philippines (Figure 2a). The non-native *C. revoluta* is widely cultivated throughout most inhabited Philippine islands [43], and an inadvertent invasion of *L. pandava vapanda* within urban settings may have occurred in the past when *C. revoluta* plants were imported to support the nursery trade.

Despite substantial diversity of geographic origin and subspecies among the various feeding trials, every butterfly population obtained from in situ *Cycas* hosts exhibited unambiguous food selection behavior. Contrarily, every urban butterfly population that was studied exhibited no discrimination behaviors in food selection and consumed any *Cycas* leaflet that was offered ad libitum. Based on these results, the ability to discriminate during binary choices and feed primarily on high quality food was restricted to the wild populations.

The feeding efficiency determined as time lapsed until feeding was initiated was remarkably different for the wild type versus the urban type butterflies. In every paired experiment, the urban population initiated feeding with greater speed, averaging 0.8 min; and the wild population initiated feeding more slowly, averaging 2.0 min. A third difference between the wild and urban populations was measured as total leaflet area consumed during the 8 h feeding trials. In every case, the urban population (mean 288 mm^2^) consumed more than the wild population (mean 258 mm^2^), representing 12% greater daily consumption potential for the urban butterflies. Based on these results, the ability to exploit all available food sources and consume greater quantities of food per day would allow the urban populations to out-compete the wild populations at the larval stage.

### 4.2. Botanic Gardens

Ex situ cycad germplasm gardens are sometimes located nearby in situ populations of native congeneric cycad species. These managed gardens containing non-native species are artificially sympatric with the native congeneric species, some of which may be sexually compatible, and therefore create new biological interactions between the garden plants and the surrounding in situ plants. For example, the Montgomery Botanical Center curates an extensive ex situ *Zamia* collection [44] and is surrounded by in situ *Zamia integrifolia* L.f. populations. The habitat was relied upon for some of the initial studies that laid the foundation for our contemporary understanding of cycad pollination [45,46,47]. Similarly, the cycad germplasm gardens curated by the University of Guam are located amongst in situ *C. micronesica* habitats. This anthropogenic creation of close proximity between ex situ and in situ congenerics may cause consequential interactions through insects which travel between the garden and natural plants.

The permeability of botanic garden boundaries (e.g., [48]) influences herbivory in locations where a native cycad species resides. First, the non-native germplasm in botanic gardens may serve as anthropogenic brood sites of *L. pandava,* magnifying the neighborhood herbivory pressures imposed on the adjacent native *Cycas* host plants simply by increasing the butterfly population size. This same phenomenon may occur where non-native *Zamia* species in botanic gardens act as brood sites for *Eumaeus atala* Poey, magnifying the herbivory pressure on surrounding native *Zamia* plants. Second, the feeding behaviors of larvae on an in situ native host plant may originate from an egg oviposited by an adult that was reared on non-native botanic garden host plants. The results herein indicate these larvae from a garden-reared adult may cause greater herbivory damage than an oligophagous larvae originating from the native butterfly population. Third, an adult butterfly that was reared on high quality non-native host species inside the botanic garden may cause more in situ damage because of greater longevity and fecundity of these adults [17]. Finally, a more insidious threat may occur when the non-native cycad germplasm in a botanic garden becomes the source of genetic pollution of surrounding native cycad species due to the efflux of pollinator species carrying non-native but sexually compatible pollen to the native plants. The resulting hybrid zone surrounding the botanic garden perimeters would likely receive more diverse genetic pollution and expand in geographic range over time. Clearly, cycad germplasm curators are bound by an ethical responsibility to better understand how to protect the surrounding in situ native plants from the consequences of flying insects which move in and out of the managed ex situ plants.

One facet of garden management that cycad conservationists may exploit is the use of beneficial mixtures of host species to develop neighborhood diversity patterns that beneficially influence herbivore behavior. These protocols may clarify the role of plant genotypic diversity for modifying pest behaviors within the garden settings and decrease the impact that the garden has on the surrounding ecosystem [49,50,51,52]. A pragmatic outcome of this agenda may occur when insecticide applications are used in managed botanic gardens. The *Cycas* species which exhibit minimal *L. pandava* damage are clearly not in need of as much effort in applying expensive pesticides [15]. The costs of the pesticide program may therefore be lessened by grouping the heavily damaged *Cycas* species in close proximity as a means of reducing labor costs and increasing efficacy of the targeted pesticide applications.

### 4.3. Limited Focus on Relevant Concepts

The reasons for the more rapid larval growth and greater fecundity of adults when *L. pandava* are reared on the *Cycas* species that were considered to be high quality are not understood. This knowledge gap limits our ability to best define conservation protocols for host species in newly invaded territories and the reasons for differences in quality deserve further study.

Climate may be an important driver of *L pandava* herbivory in some locations, but the influences of climate change on *L. pandava* behavior has not been extensively discussed. Seasonal differences in *L. pandava* damage and have been reported in monsoon climates where increases in *L. pandava* herbivory occur during seasons with less rainfall [16,53]. Moreover, seasonal fluctuations of temperature may influence *L. pandava* performance [54]. These findings indicate future climate change may directly influence the threat levels of *L. pandava* on cycad conservation, yet what is known is not enough to make predictions about these phenomena. Seasonal difference in *L. pandava* population pressures in tropical regions such as Guam and the Philippines may be mediated by way of the timing and frequency of vegetative growth events which are required for *L. pandava* regeneration.

The issue of biological control has also not been developed for countering *L. pandava* damage to *Cycas* plants through management decisions. The potential for biological control of *L. pandava* has been discussed [55,56], and clearly needs to be afforded direct empirical research attention to create a sustainable approach to addressing the herbivory threat.

The topic of multiple cycad-specific arthropod herbivore invasions has also been inadequately studied. Indeed, *Cycas* plants are not host for single herbivore species, contrarily they may be required to withstand damage from multiple non-native herbivores representing contrasting guilds [57]. One recent study illuminated the relatively greater threat to *C. micronesica* imposed by *A. yasumatsui* when compared with several other herbivores including *L. pandava* [58]. These outcomes were evident in Guam Island where the *A. yasumatsui* invasion preceded the *L. pandava* invasion, but equally evident in Rota Island where the *L. pandava* invasion preceded the *A. yasumatsui* invasion. The butterfly and armored scale populations appear to be in direct concurrent competition, as the damage by *L. pandava* increased and decreased in tandem with decreased and increased *A. yasumatsui* damage [57]. Developing the best management protocols to combat these and other unique interactions among multiple invasive cycad pests will require more research.

Finally, continuing expansion of the invasive range of this devastating cycad pest calls for greater focus on inspection and quarantine protocols in all state territories with a native *Cycas* species. The first record of a new country invasion was reported in 1992 when the island of Okinawa was reported as invaded [59]. The most recent record of invasion was 2025 when regenerating *L. pandava* populations were reported in Australia [60]. These new invasions are particularly threatening to range-limited endemic *Cycas* species that have never been challenged by an alien leaf herbivore. However, the threats to native biota may be even more magnified for *Cycas* populations that support a native leaf herbivore, such as the Australian Cycad Blue *Theclinesthes onycha* Hewitson [61]. In these situations, the threat of extinction is caused by *L. pandava* is not restricted to the new *Cycas* host species, it extends to the native herbivore which is forced into direct competition with the invasive species, as the native butterfly relies on the *Cycas* host for regeneration.

## 5. Conclusions

The threat to cycad conservation caused by the cycad leaf herbivore *L. pandava* has increased in recent years due to new territory invasions. The results from dual choice cafeteria trials indicated that *L. pandava* larvae derived from in situ *Cycas* habitats exhibited beneficial discriminatory behaviors when challenged with novel food choices. However, these selection behaviors were not evident for larvae from a resident *L. pandava* population which was provisioned with larval food from numerous *Cycas* species that were not available within the butterfly’s native habitat. The findings indicate that *Cycas* plants within botanic gardens with resident *L. pandava* are threatened by an herbivore that is more voracious than the corresponding wild type herbivore. Moreover, when a botanic garden is located within the natural range of a native *Cycas* species, or when an insular *Cycas* population experiences a new *L. pandava* invasion, the threats to the native plants are magnified by the herbivore’s ability to become less discerning, more efficient, and more voracious than the wild type butterflies.

## Figures and Tables

**Table 1:** Experimental conditions for *Luthrodes pandava* larvae feeding trials using *Cycas* hosts in Guam, Philippines, and Thailand.

Trial	Dates	Max Temp (°C)	Min Temp (°C)	PPFD (µmol·m^−2^·s^−1^)
Thailand *C. nongnoochiae*	19–24 July 2013	32.9	26.6	24–32
Philippine *C. edentata*	30 January–5 February 2019	28.7	22.3	33–40
Guam Population	29 January–4 February 2023	30.1	26.1	20–24
Philippine *C. zambalensis*	28 May–5 June 2024	33.3	26.1	26–31

## Data Availability

The original contributions presented in this study are included in the article. Further inquiries can be directed to the corresponding author.

## Abbreviations

The following abbreviations are used in this manuscript:

NNTBG	Nong Nooch Tropical Botanical Garden
